# Different associations of white matter lesions with depression and cognition

**DOI:** 10.1186/1471-2377-12-83

**Published:** 2012-08-25

**Authors:** Jun-Young Lee, Philip Insel, R Scott Mackin, Norbert Schuff, Helena Chui, Charles DeCarli, Kee Hyung Park, Susanne G Mueller, Michael W Weiner

**Affiliations:** 1 Center for Imaging of Neurodegenerative Diseases, Veterans Affairs Medical Center, 4150 Clement Street, San Francisco, CA 94121, USA; 2 Department of Psychiatry, Seoul National University Boramae Hospital, Seoul, Korea; 3 Department of Psychiatry, University of California, San Francisco, CA, USA; 4 Department of Radiology and Biomedical Imaging, University of California, San Francisco, CA, USA; 5 Department of Neurology, University of Southern California, Los Angeles, CA, USA; 6 Department of Neurology and Center for Neuroscience, University of California, Davis, Sacramento, CA, USA; 7 Department of Neurology, Gachon University Gil Hospital, Incheon, Korea; 8 Department of Radiology, Psychiatry, Neurology, and Medicine, University of California, San Francisco, CA, USA

**Keywords:** Leukoaraiosis, Depression, Cognition, Frontal lobe, Mediation

## Abstract

**Background:**

To test the hypothesis that white matter lesions (WML) are primarily associated with regional frontal cortical volumes, and to determine the mediating effects of these regional frontal cortices on the associations of WML with depressive symptoms and cognitive dysfunction.

**Methods:**

Structural brains MRIs were performed on 161 participants: cognitively normal, cognitive impaired but not demented, and demented participants. Lobar WML volumes, regional frontal cortical volumes, depressive symptom severity, and cognitive abilities were measured. Multiple linear regression analyses were used to identify WML volume effects on frontal cortical volume. Structural equation modeling was used to determine the MRI-depression and the MRI-cognition path relationships.

**Results:**

WML predicted frontal cortical volume, particularly in medial orbirtofrontal cortex, irrespective of age, gender, education, and group status. WML directly predicted depressive score, and this relationship was not mediated by regional frontal cortices. In contrast, the association between WML and cognitive function was indirect and mediated by regional frontal cortices.

**Conclusions:**

These findings suggest that the neurobiological mechanisms underpinning depressive symptoms and cognitive dysfunction in older adults may differ.

## Background

White matter lesions (WML) are commonly observed on T-2 weighted MRI of elders and pathologically consistent with loss of myelin and axons due to incomplete infarction and/or cerebral aging. WML have been found in 11-20% of the old-age population without dementia and have been observed in almost all elders over age 85 [1].

Although several studies reported an association between WML volume and global cortical atrophy [2,3], the extent to which WML are related to regional brain atrophy remain uncertain. In order to understand the interaction between Alzheimer pathology and presumptive cerebrovascular pathology, most of regional studies [4,5] examined the relationship between WML and structures of the mesial temporal lobe, such as the hippocampus, entorhinal cortex, and medial temporal atrophy. In addition, the frontal lobe is a region of especial interest because of its possible relationship to both mood symptoms [6-8] and cognitive impairment shown in cerebral small vessel disease [5,9-11]. Therefore, the first goal of this study is to test the hypothesis that WML are primarily associated with frontal cortical volume and we sought to determine the specific regions of frontal cortex which are associated with WML.

Several studies also have shown that both WML and frontal cortex are associated with depressive symptom severity [6-8] and impaired cognitive functions which are primarily characterized by memory and executive dysfunction in older adults [5,9-11]. However, the specific associations among WML, regional frontal cortices, depressive symptoms and/or cognitive dysfunction have not been clearly surveyed. If WML are associated with regional frontal cortical volume, WML could be related to depression, cognitive impairment, or both, either directly or via cortical volume. Therefore, the second goal of this study is to determine the mediation effect of regional frontal cortices on the association of WML with depressive symptoms and/or cognitive impairment.

## Methods

### Participants

Subjects were comprised of subjects recruited from 3 academic dementia centers (University of California Davis, University of California San Francisco, and University of Southern California) as part of a multicenter collaborative study examining contributions of subcortical ischemic vascular dementia and Alzheimer’s disease to cognitive impairment in older adults. Exclusion criteria included age younger than 55 years, non-English speaking, severe dementia (Clinical Dementia Rating (CDR) > 2), evidence of alcohol or substance abuse, head trauma with loss of consciousness lasting longer than 15 minutes, severe medical illness, neurologic or psychiatric disorders other than dementia, or currently taking medications likely to affect cognitive function. Severely depressed patients who had recurrent suicidal ideas or severe impairments in social function were also excluded. In addition, subjects were excluded if the MRI showed evidence of cortical infarction, hemorrhage, or structural brain disease other than atrophy, lacunes, or WML.

Participants were grouped by the levels of cognitive impairment as normal cognition control (CDR = 0), cognitively impaired but not demented (CIND) (CDR = 0.5), and demented (CDR ≥ 1). Alzheimer’s disease were diagnosed using National Institute of Neurologic and Communicative Disorders and Stroke and the Alzheimer's Disease and Related Disorders Association (NINCDS-ADRDA) [12] diagnostic criteria and ischemic vascular dementia were diagnosed by California Alzheimer's Disease Diagnostic and Treatment Centers (ADDTC) [13] criteria.

The study had been reviewed and approved by the institutional review boards of all study sites and written informed consent had been obtained from the study participants or their legal representatives.

### Neuropsychological tests

All participants received a standardized battery of neuropsychological tests. The composite scores were calculated using item response theory methods and transformed to a standard scale with a mean of 100 and a SD of 15 [14]. Verbal memory score was calculated based on the Word List Learning tasks of the Memory Assessment Scales, visual memory score on the tasks of the Biber Figure Learning Test and executive score on the Initiation-Perseveration task from the Mattis Dementia Rating Scale, the letter fluency task, the digit span backward and the visual span backward tasks from the Wechsler Memory Scale-Revised. Every subject received clinical interview for depression by clinical psychologists using Minimum Uniform Data Set (MUDS) structured interview [15]. Depressive score was defined as the number of depressive symptoms meeting the nine criterion symptoms by Diagnostic and Statistical Manual of Mental Disorders, third edition, revised (DSM-IIIR) [16]. The number of depressive symptoms based on DSM criteria reflects severity of disease well [17]. The CDR and the Mini Mental State Examination were also used.

### MRI data acquisition and processing

The entire brain was imaged using a 1.5 T Magnetom VISION system (Siemens, Erlangen, Germany) equipped with a standard quadrature head coil. We derived WML volume from proton density (PD)-weighted (PD) and T2-weighted spin-echo axial images from a double spin-echo sequence (repetition time (TR)/echo time (TE)1/TE2 = 2500/20/80 msec; 1.0 × 1.4 mm^2^ in-plane resolution; slice thickness 3 mm oriented along the AC-PC line) and regional frontal gray matter volumes from T1-weighted coronal three dimensional images from the magnetization-prepared rapid gradient Echo (MPRAGE) (TR/TE/TO = 13.5/7/300 msec; 1.0 × 1.0 mm^2^ in plane resolution; slice thickness 1.4 mm oriented along the long axis of the hippocampus).

Total WML volume and total intracranial volume (TIV) were based on the multi-channel segmentation of Expectation Maximization Segmentation [18] and lobar WML segmentation were based on the atlas-based deformable registration method [19]. The technical details of these procedures are described in prior publication [11]. Lacunes were defined as small (> 2 mm) areas of subcortical gray and white matter with increased signal relative to Cerebrospinal fluid (CSF) on proton density MRI.

Frontal regional cortical volumes were based on the Freesurfer image analysis software 4.0 version (http://surfer.nmr.mgh.harvard.edu/). The technical details of these procedures are described in prior publication [20]. Cortical volume combined both thickness and area information and parcellation of the cerebral cortex into units based on gyral and sulcal structure [21] were done to get regional frontal volumes.

### Statistical analysis

To account for variations in head sizes, all gray matter volumes and WML volume were normalized to each TIV according to: VOLn = VOLr × TIVm/TIVr. Here VOLn and VOLr are the normalized volume and the raw volume of a subject, respectively; and TIVm and TIVr are the mean TIV from all subjects and the TIV of the subject, respectively. Volume unit is cm^3^. WML volumes were log-transformed to achieve normal distribution. We did same analysis in subjects who had not lacunar infarcts to test these relationships without the effect of lacunes.

We used multiple linear regression models. Independent variables were WML volume, age, gender, years of education, and groups and dependent variable was each lobar cortical volume or each regional frontal cortical volume. The interaction between WML and group status on predicting frontal cortical volume was also tested in the same linear regression model by examining the difference of regression coefficients between WML volume and frontal gray matter volume among control, CIND subjects, and dementia patients. Plots of residuals were used to evaluate model assumptions. Residuals appeared normal and linear regression assumptions were satisfied. Regression coefficients were standardized by all variables centered on the mean and scaled by the standard deviation to compare them easily.

**Figure 1:**
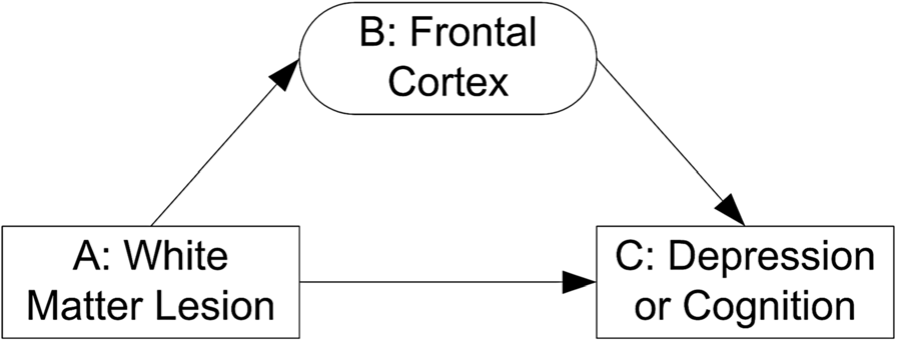
Path diagram for the mediation model.

We employed the structural equation modeling (SEM) to test for the mediation effect of frontal cortex on WML association with depressive symptoms or cognitive function. Mediation means that independent variable (A: WML) causes an intervening variable (B: frontal cortex), which in turn causes the dependent variable (C: depression or cognition) [22] (Figure1). Four conditions must be met for B to be a mediator: 1) A is significantly associated with C; 2) A is significantly associated with B; 3) B is significantly associated with C after controlling for A; 4) the impact of A on C is significantly less after controlling for B [23]. We used SEM to test these conditions because we had multiple measures for frontal cortex and cognition [23]. We reported model fitness by the χ^2^, the comparative fit index (CFI), and the root mean-square error of approximation (RMSEA). An excellent fitting model is usually indicated by a non-significant χ^2^, CFI >0.90, and RMSEA <0.1 [24]. Path coefficients were standardized by subtracting the sample mean and then dividing by the sample SD. The Amos Version 18.0 software (SPSS, Chicago, Illinois) was used for all SEM analyses utilizing maximum likelihood estimation. The Kruskal-Wallis test was used to test for mean differences among groups. A p value less than 0.05 was considered significant.

## Results

### Demographic and clinical characteristics

Table1 shows demographic data, results of cognitive tests, and MRI volumes for all 161 subjects. The mean age of subjects was 74.4 (SD = 7.8) and the mean years of education was 14.9 (SD = 3.2). 56 percent of participants were male (n = 90). Sixty-four subjects had normal cognition, 44 subjects were CIND, and 53 subjects were diagnosed with dementia. Thirty-four of the demented subjects were diagnosed with Alzheimer’s disease, 9 with mixed Alzheimer's disease and subcortical ischemic vascular dementia, and 10 with subcortical ischemic vascular dementia. Seventy-three subjects had no lacune (41 in normal controls, 12 in cognitively impaired but not demented subjects, 20 in dementia subjects). Twenty-five subjects were diagnosed with current major depressive disorder or had been diagnosed with previous major depressive disorder (4 in normal controls, 11 in cognitively impaired but not demented subjects, 10 in dementia subjects).

**Table 1:** Clinical characteristics of the study population

	**All (n=161)**	**Control (n=64)**	**CIND (n=44)**	**Dementia (n=53)**	**p**
M/F	90/71	29/35	30/14	31/22	0.06
Age, y	74.4 (7.8)	73.0 (7.0)	73.0 (7.9)	77.1 (8.1)	0.006
Education, y	14.9 (3.2)	15.5 (3.0)	15.4 (3.0)	13.7 (3.3)	0.002
MMSE	26.6 (4.3)	29.2 (1.0)	27.9 (1.7)	22.4 (5.2)	<0.001
Depressive score	1.5 (1.7)	0.5 (1.0)	1.8 (0.5)	2.4 (1.9)	<0.001
Verbal memory score	88.3 (24.0)	106.7 (15.8)	87.5 (15.3)	62.6 (15.1)	<0.001
Visual memory score	86.6 (22.4)	102.9 (12.1)	85.0 (18.3)	61.6 (14.5)	<0.001
Executive score	90.8 (18.6)	100.0 (13.8)	90.9 (17.5)	75.2 (16.7)	<0.001
Frontal gray matter volume, cm^3^	200.7 (13.8)	206.7 (12.7)	200.6 (12.4)	193.4 (13.0)	<0.001
White matter lesion volume, cm^3^	19.6 (17.7)	14.0 (6.9)	19.7 (15.6)	26.2 (23.8)	<0.001

Mean (standard deviation). All gray matter volumes and WML volume were normalized to total intracranial volume. CIND, Cognitively Impaired Not Demented; MMSE, Mini-Mental State Exam.

### WML and frontal cortical volume

**Figure 2:**
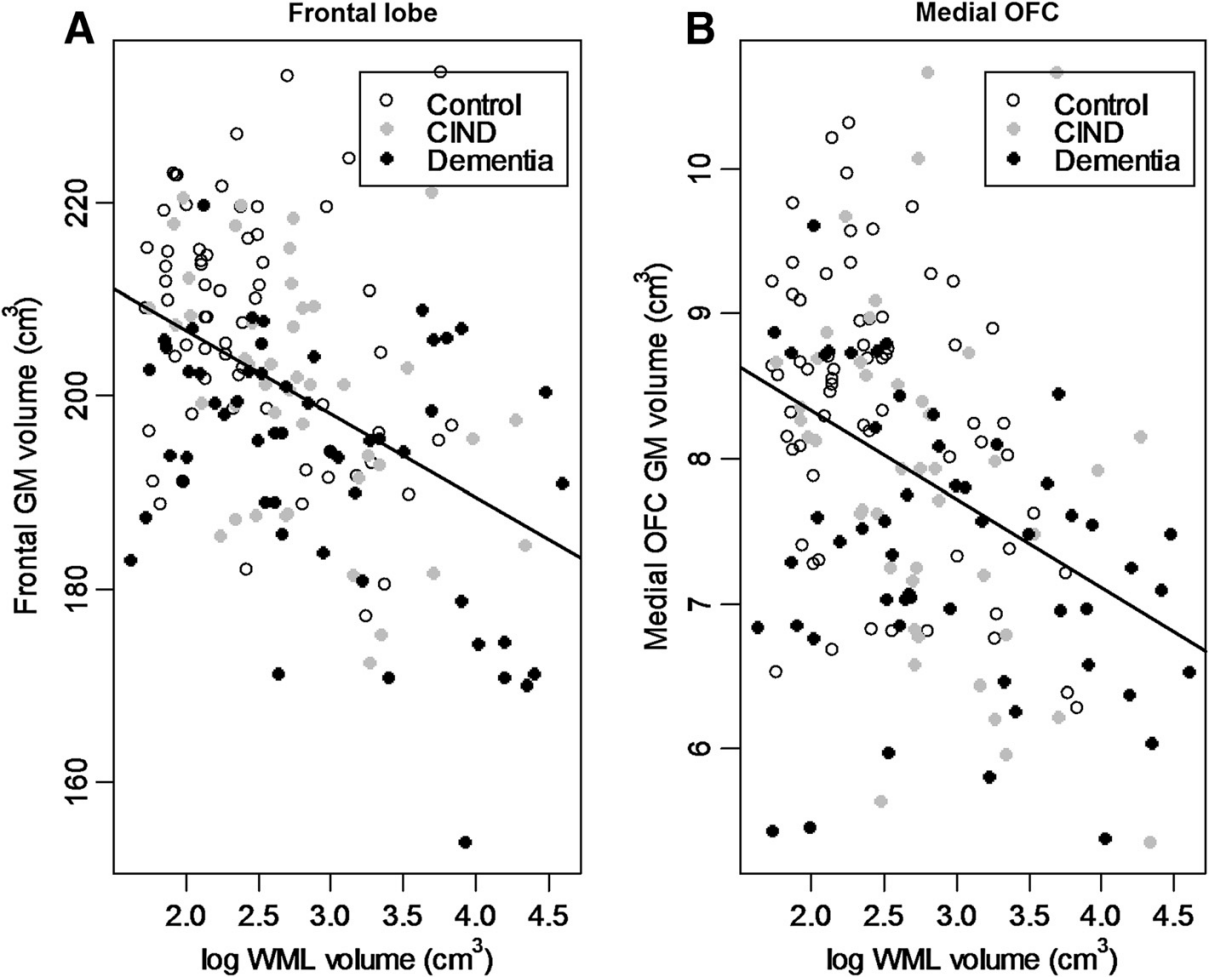
**Relationship of white matter lesion (WML) volume with A. Frontal gray matter (GM) volume (β = −0.24, SE = 0.07, p = 0.002), B. Medial orbitofrontal (OFC) GM volume (β = −0.16, SE = 0.07, p = 0.04) in all groups. Independent variables are log-transformed WML volume, age, gender, years of education, and group status.** β is the standardized regression coefficient.

We conducted linear regression to determine the relationship between total WML volume and each lobar cortical volume after controlling the effects of age, gender, years of education and groups. Total WML predicted only frontal cortical volume (standardized β = −0.24, SE = 0.07, p = 0.002) (Figure2) and did not predict other lobar cortical volumes. There was no interaction between group status and WML volume in predicting frontal cortical volume (F (2, 154) = 0.64, p = 0.53). It indicated that group status did not affect the association between WML and frontal cortical volume. In subjects who had no lacune, total WML predicted only frontal cortical volume, too (standardized β = −0.24, SE = 0.08, p = 0.002).

All lobar WML volumes [frontal WML (β = −0.24, SE = 0.08, p = 0.003); temporal WML (β = −0.14, SE = 0.07, p = 0.05); parietal WML (β = −0.27, SE = 0.07, p < 0.001); and occipital WML (β = −0.15, SE = 0.07, p = 0.04)] predicted frontal cortical volume.

We conducted linear regression to determine the relationship between total WML volume and each regional frontal cortical volume after controlling the effects of age, gender, years of education and group status (Table2). Among regional frontal cortices, only medial orbitofrontal cortical volume (β = −0.16, SE = 0.07, p = 0.04) was significantly associated with total WML (Figure2). Pars opercularis (β = −0.15, SE = 0.08, p = 0.08) and precentral gyrus (β = −0.14, SE = 0.08, p = 0.08) showed a trend to be associated with total WML. In subjects who had no lacune, only medial orbitofrontal cortical volume was significantly associated with total WML, too (β = −0.16, SE = 0.08, p = 0.04).

**Table 2:** Prediction of regional frontal gray matter volumes by white matter lesion (WML) volume

	**Log WML volume**		
**Dependent variable**	**β**	**SE**	**p**
Medial orbitofrontal cortex	−0.16	0.07	0.04
Lateral orbitofrontal cortex	−0.07	0.08	0.40
Rostral anterior cingulated cortex	−0.11	0.08	0.18
Caudal anterior cingulated cortex	−0.11	0.09	0.20
Frontal pole	−0.07	0.09	0.43
Superior frontal gyrus	−0.09	0.08	0.27
Rostral middle frontal gyrus	−0.04	0.08	0.65
Caudal middle frontal gyrus	−0.11	0.08	0.17
Pars opercularis	−0.15	0.08	0.08
Pars orbitalis	−0.01	0.09	0.88
Pars triangularis	−0.09	0.08	0.26
Precentral gyrus	−0.14	0.08	0.08

Independent variables are log-transformed WML volume, age, gender, years of education, and group status and dependent variable is each regional frontal gray matter volume. β is the standardized regression coefficient.

### WML, frontal cortex, and depressive symptoms

**Figure 3:**
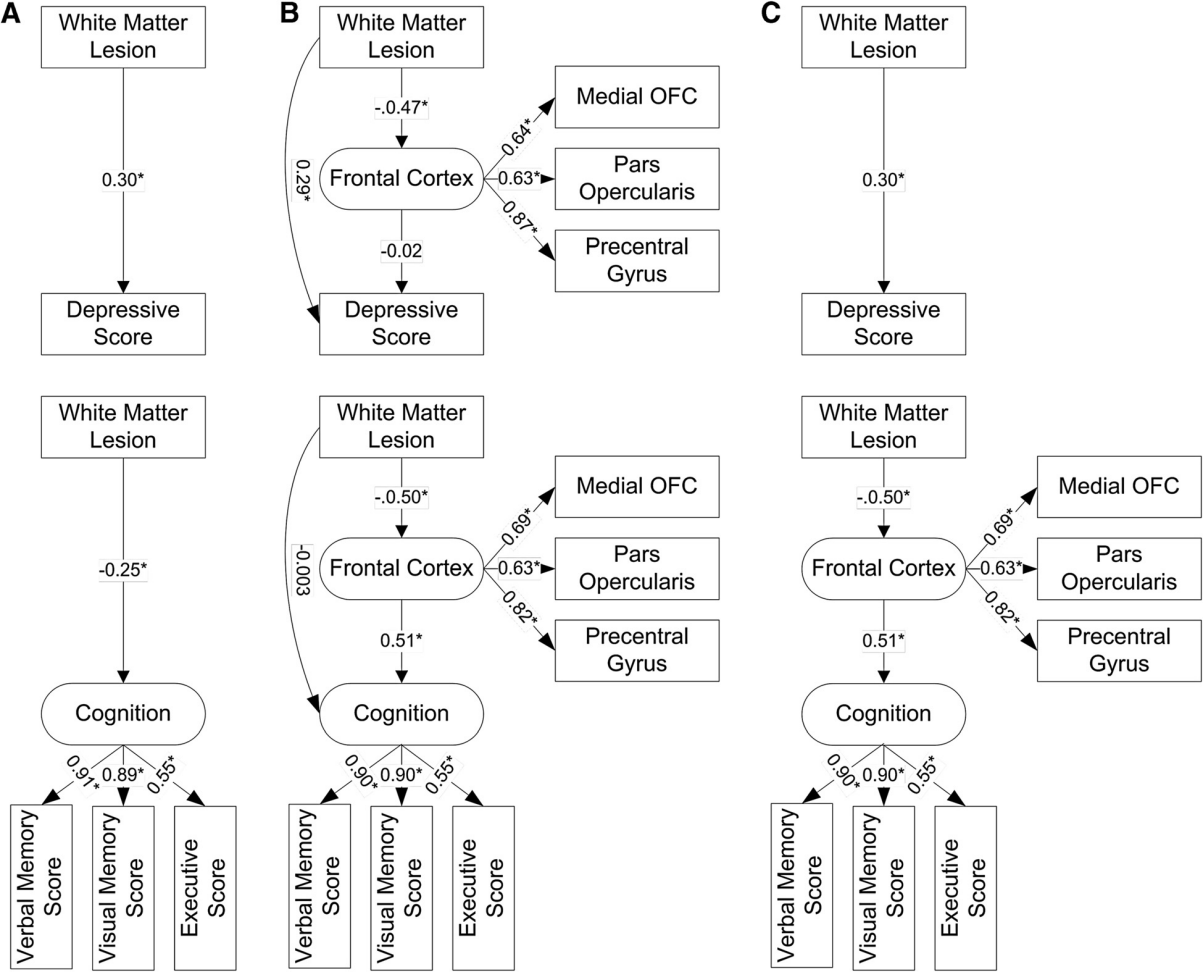
**Structural equation models with standardized path coefficients (β) showing the MRI-depression and MRI-cognition path in A. the direct effect model, B. the mediation model, and C. the final model.** In ‘**A**’ model, white matter lesion (WML) predicted both depressive score (β = 0.30, SE = 0.08, p < 0.001) and cognition (β = −0.25, SE = 0.09, p = 0.01). In ‘**B**’ model, the latent variable ‘frontal cortex’ was added as a mediator. Note that frontal cortex (β = − 0.02, SE = 0.10, p = 0.85) did not predict depressive score and WML volume (β = −0.003, SE = 0.09, p = 0.97) did not predict cognition directly. Therefore, in ‘**C**’ Model, we removed frontal cortex in MRI-depression path and the direct path from WML to cognition in MRI-cognition path. Note that WML predicted depressive score directly but predicted cognition through frontal cortex. Circles represent latent variables, and rectangles represent measured variables. Estimated error terms were omitted to simplify the figures. *p < 0.05.

Because total WML predicted frontal cortical volume atrophy, we used SEM to test relationship among WML volume, frontal cortex, and depressive symptoms (Figure3). Frontal cortex was defined as a latent variable and constructed by three indicators which had a trend to be associated with WML: medial orbitofrontal cortical volume, pars opercularis cortical volume, and precentral gyral cortical volume. All three indicators predicted frontal cortex significantly (p < 0.001).

In the direct effect model, we included total WML volume as a predictor and depressive score as a dependent variable. WML predicted depressive score (β = 0.30, SE = 0.08, p < 0.001) significantly.

In the mediation model, we added frontal cortex as a mediator between total WML and depressive score. This model showed an excellent fit to the data (χ^2^ = 4.4, df = 4, p = 0.36, CFI = 0.99, RMSEA = 0.03) and total WML predicted frontal cortex (β = −0.47, SE = 0.12, p < 0.001) and depressive score directly (β = 0.29, SE = 0.10, p = 0.003) but frontal cortex did not predict depressive score (β = −0.02, SE = 0.10, p = 0.85). It indicated WML - depression path was direct. When WML - depression path were constrained to zero, χ^2^ change was significant (χ^2^ change = 8.0, df change = 1, p < 0.01). It indicated WML - depression path was not mediated by frontal cortex. Therefore, we adopted the direct WML - depression effect model as a final depression predicting model (Figure3). In subjects who had no lacune, the direct WML - depression effect model was also well-fitted.

In multiple linear regression controlling for age, gender, years of education, and group status, among all regional frontal cortex and all lobar WML, depressive score was also only predicted by lobar WML: frontal WML (β = 0.26, SE = 0.09, p = 0.003) and temporal WML (β = 0.22, SE = 0.08, p = 0.005).

### WML, frontal cortex, and cognitive function

We used SEM to test relationships among total WML volume, frontal cortex and cognitive function (Figure3). Cognition was defined as a latent variable and constructed by three indicators: verbal memory score, visual memory score, and executive score. All indicators predicted cognition significantly (p < 0.001).

In the direct effect model, we included WML volume as a predictor, cognition as a dependent variable. Total WML predicted cognition (β = −0.25, SE = 0.09, p = 0.01) significantly.

In the mediation model, we added frontal cortex as a mediator between total WML and cognition. This model showed a good fit to the data (χ^2^ = 20.7, df = 12, p = 0.06, CFI = 0.97, RMSEA = 0.08). Total WML significantly predicted frontal cortex (β = −0.50, SE = 0.10, p < 0.001) but did not predict cognition directly (β = 0.003, SE = 0.09, p = 0.97). Frontal cortex predicted cognition (β = 0.51, SE = 0.13, p < 0.001) significantly. It indicated the effect of total WML on cognition was indirect. When WML - cognition path were constrained to zero, χ^2^ change was not significant (χ^2^ change = 0.1, df change = 1, p > 0.05). It indicated WML - cognition path was mediated by frontal cortex. Because direct WML - cognition path was not significant, we adopted the mediation model without direct WML - cognition path as a final cognition predicting model. In subjects who had no lacune, the mediation model without direct WML - cognition path was also well-fitted.

In multiple linear regression, cognition was associated with regional frontal cortex and lobar WML: verbal memory score was predicted by medial orbitofrontal cortex (β = 0.11, SE = 0.05, p = 0.05), superior frontal gyrus (β = 0.11, SE = 0.05, p = 0.05), temporal WML (β = 0.11, SE = 0.05, p = 0.05), and parietal WML (β = 0.13, SE = 0.06, p = 0.03); visual memory score was predicted by medial orbitofrontal cortex (β = 0.15, SE = 0.06, p = 0.01) and lateral orbitofrontal cortex (β = 0.14, SE = 0.06, p = 0.02); and executive score was predicted by precentral gyrus (β = 0.16, SE = 0.08, p = 0.04).

## Discussion

The major findings of this study are: 1. WML predicted frontal cortical volume, particularly the medial orbitofrontal cortical volume among all groups after controlling TIV, age, gender, years of education, and the presence of lacune; 2. WML predicted depressive symptom severity directly and this relationship was not mediated by regional frontal cortical volume; 3. The relationship between WML and cognitive function was indirect and mediated by regional frontal cortex. These findings confirm the previous result from this collaborative project reporting that WML are related to depression and gray matter volume is related to cognition [11]. Our study has important implications for understanding the potential mechanisms underlying these dissociations. Each of these findings will be discussed below.

The first major finding of this study is that all lobar WML are associated with frontal cortical volume irrespective of groups. This association is strongest with medial orbitofrontal cortex. Our findings are consistent with one previous functional MRI study showed that WML were associated with decreased frontal activation [25] in healthy elders and one previous FDG PET study showed that WML predicted decreased frontal glucose metabolism in all cognitive groups [26], however, these studies did not explore specific associations with orbitofrontal cortex.

WML can be related to the frontal cortical atrophy because WML and frontal cortical atrophy both are related to aging, ischemic damage, and cortical degenerative changes. In frontal lobes, lateral and orbitofrontal regions in frontal cortex have been reported to be prominently affected by cerebral aging among elders [27,28]. Thus, the selective volume loss of medial orbitofrontal cortex associated with WML could be explained by cerebral aging. However, we found that WML volume was related to regional frontal cortical volume after controlling for age, and we thus conclude that this relationship is not explained solely by normal aging. It is not clear that the ischemic damage to the axons of subcortical white matter causes retrograde degeneration of neuronal bodies in frontal cortex or degenerative neuronal cell death of frontal cortex causes anterograde Wallerian degeneration of the axons of subcortical white matter. Longitudinal study will be needed to find the causal relationship between subcortical WML and frontal cortical atrophy.

The second major finding of this study is that WML, especially frontal and temporal WML, are directly related to depressive symptom severity and frontal cortex do not mediate this association.

Previous studies have also reported that geriatric depression is associated with WML [6-8]. Depressive symptoms in elders consist of diverse cognitive, emotive, and physiological symptoms. Therefore, geriatric depression can be caused by abnormalities of brain connections rather than a single brain area abnormality and WML association with decreased anisotropy and reduced myelin integrity of white matter tracts in frontal lobes [29] indicates the possibility of disruption of white matter tracked by WML.

In this study, depressive symptom severity is not associated with regional frontal cortices. However, some other studies have reported that geriatric depressive disorder is associated with orbitofrontal cortical atrophy in elders [30,31], but these studies did not consider WML effect on depression. Our findings may be in part due to our sample criteria which exclude severe depression because prefrontal cortical atrophy was reported to be associated with severe depression and not to mild depression [32].

The third major finding of this study is that WML are related to impaired cognitive function but the relationship between WML and impaired cognitive function was indirect and mediated by volume of frontal cortex. Orbitofrontal cortex is commonly related to memory encoding [33] and to apathy which can influence the executive performance negatively [34]. Therefore, orbitofrontal cortical atrophy can cause cognitive impairment including memory and executive dysfunction.

Previous studies [9,10,35] which reported the relationship of WML to cognitive function usually did not consider the role of cortical atrophy. In several studies there was no direct association between WML and cognitive function once global cortical atrophy was accounted for [3,11,36,37]. These findings add to the view that cognitive dysfunction related to WML may be result of the frontal cortical atrophy secondary to WML rather than the direct result of WML.

Several features of this study differed from previous reports. First, we measured WML volume and regional cortical volumes instead of ordinal scoring system. There were numerous visual WML rating scales and these subjective scales displayed ceiling effects and showed poor sensitivities [38]. Therefore, volumetric measurement of WML and other cortical areas may yield more accurate results. Second, we used SEM to explore the mediation effect of frontal cortex. SEM has several advantages. We can have multiple indicators for each construct [23] and multiple indicators do not cause collinearity problems differently to linear regression model [37].

We have several limitations. These results are from a cross-sectional data and therefore causality between WML and atrophy cannot be confirmed. We do not divide WML into periventricular WML and deep WML, so we cannot analyze the effect of WML sites on cognition and depression. We excluded severely depressed patients who had recurrent suicidal ideas or severe impairments in social function. Therefore, we cannot rule out the possibility of the association between frontal lobes and severe depression. Due to relative small sample size, we cannot control vascular risk factors and drug effects.

## Conclusions

WML volume predicted frontal cortical volume atrophy, especially medial orbitofrontal cortex. Depressive symptom severity are directly associated with WML but cognitive impairment was indirectly associated with WML and this association was mediated by frontal cortical volume. These findings suggest that neurobiological mechanisms of depressive symptoms and cognitive dysfunction by WML may be different. Depression may result from diminished input from subcortical noradrenergic and serotonergic pathways due either to subcortical neurodegeneration or disruption by WML. In contrast, cognitive impairment may be driven more by primary neocortical synaptic degeneration or secondary cortical degeneration by aging process related to WML rather than disruption of axonal projections.

## Abbreviations

WML: White matter lesions; CDR: Clinical dementia rating; CIND: Cognitively impaired but not demented; TIV: Total intracranial volume; CSF: Cerebrospinal fluid; DSM: Diagnostic and statistical manual of mental disorders; SEM: Structural equation modeling.

## Competing interests

The authors declare that they have no competing interests regarding this study. This study was supported by a grant from the NIH (P01AG012435) and the National Research Foundation of Korea Grant funded by the Korean Government MEST, Basic Research Promotion Fund (NRF-2011-013-E00027).

## Authors’ contributions

JYL, PI, RSM, NS, HC, CD, KHP, SGM, and MWW have made substantial contributions to conception and design, or acquisition of data, or analysis and interpretation of data. JYL, PI, NS, and MWW have been involved in drafting the manuscript, while RSM, HC, CD, KHP, SGM revised it critically for important intellectual content. All authors have given final approval of the version to be published.

## Pre-publication history

The pre-publication history for this paper can be accessed here:

http://www.biomedcentral.com/1471-2377/12/83/prepub
